# Low-calorie diets for people with isolated impaired fasting glucose

**DOI:** 10.1038/s43856-024-00466-2

**Published:** 2024-03-01

**Authors:** Sathish Thirunavukkarasu, Roy Taylor, Kamlesh Khunti, Robyn J. Tapp, Anne Raben, Ruixin Zhu, Nitin Kapoor, K M Venkat Narayan, Mohammed K. Ali, Jonathan E. Shaw

**Affiliations:** 1 Department of Family and Preventive Medicine, School of Medicine, Emory University, Atlanta, GA USA; 2 Translational and Clinical Research Institute, Magnetic Resonance Centre, Campus for Ageing and Vitality, Newcastle University, Newcastle upon Tyne, UK; 3 Diabetes Research Centre, University of Leicester, Leicester, UK; 4 Centre for Intelligent Health Care, Coventry University, Coventry, UK; 5 Department of Nutrition, Exercise and Sports, Faculty of Science, University of Copenhagen, Copenhagen, Denmark; 6 Department of Endocrinology, Diabetes and Metabolism, Christian Medical College, Vellore, India; 7 Emory Global Diabetes Research Center, Woodruff Health Sciences Center, Emory University, Atlanta, GA USA; 8 Baker Heart and Diabetes Institute, Melbourne, VIC Australia

**Keywords:** Type 2 diabetes, Type 2 diabetes

## Abstract

Thirunavukkarasu et al. discuss how standard lifestyle interventions prove ineffective in preventing type 2 diabetes in individuals with isolated impaired fasting glucose, a highly prevalent prediabetes phenotype globally. They propose low-calorie diets as a promising strategy for diabetes prevention in this high-risk population.

## Teaser

Standard lifestyle interventions prove ineffective in preventing type 2 diabetes among individuals with isolated impaired fasting glucose, a highly prevalent prediabetes phenotype globally. Here, we propose low-calorie diets as a promising strategy for diabetes prevention in this high-risk population.

Prediabetes represents an intermediary stage in the development of type 2 diabetes^1^. It is characterized by elevated glucose levels that are higher than normal but below the diabetes diagnostic threshold^1^. Prediabetes is not a single condition; rather, it encompasses a diverse range of phenotypes, including isolated impaired fasting glucose (i-IFG), isolated impaired glucose tolerance (i-IGT), and IFG plus IGT^2,3^.

i-IFG constitutes a significant proportion of the global prediabetes population, ranging from 43.9% to 58.0% among Caucasians and 29.2% to 48.1% among Asian people, depending on the diagnostic criteria^4^. It is characterized by fasting hyperglycemia and normal 2-hour plasma glucose levels after a 75-g glucose load during an oral glucose tolerance test^5,6^. i-IFG is marked by impaired early-phase insulin secretion and hepatic insulin resistance (liver is less responsive to insulin action), which strongly correlates with liver fat content^2,7,8^. Individuals with i-IFG experience an annual diabetes progression rate of 3.6% to 5.2%^9^ and have a four- to six-fold higher risk of developing type 2 diabetes compared to those with normoglycemia, depending on the diagnostic criteria^9^. Additionally, i-IFG carries an elevated risk of vascular complications and all-cause mortality^3,10^.

In a systematic review by Bodhini et al. published in *Communications Medicine*, the authors investigated the variability in the effectiveness of lifestyle interventions for preventing type 2 diabetes across various sociodemographic, clinical, behavioral, and genetic factors^11^. Their analysis, based on data from 81 studies (comprising 33 unique clinical trials), demonstrated that individuals with prediabetes tend to benefit more from prevention strategies compared to those without prediabetes^11^. Consequently, the authors recommend targeting individuals with prediabetes for diabetes prevention programs. Moreover, they emphasize the importance of further research to investigate whether individuals with distinct pathophysiological features might benefit from more tailored preventive interventions. Such efforts could help address the existing gaps in evidence regarding the precision prevention of type 2 diabetes.

While standard lifestyle interventions, such as low-fat, high-fiber diets, and increased aerobic physical activity, are highly effective in reducing diabetes incidence in those with IGT, regardless of the presence of IFG, they have proven ineffective among those with i-IFG^12^. These findings stem from a recent individual participant data meta-analysis that pooled data from four randomized controlled trials conducted in India, Japan, and the UK. The analysis included 2794 participants: 1240 (44.4%), 796 (28.5%), and 758 (27.1%) had i-IFG, i-IGT, and IFG plus IGT, respectively. After a median follow-up of 2.5 years, the pooled hazard ratio for diabetes incidence in i-IFG was 0.97 (95% CI: 0.66, 1.44, *I*^*2*^ = 0), i-IGT was 0.65 (95% CI: 0.44, 0.96, *I*^*2*^ = 0), and IFG plus IGT was 0.51 (95% CI: 0.38, 0.68, *I*^*2*^ = 0); P_interaction_ = 0.01^12^. Standard lifestyle interventions primarily target the pathophysiological defects associated with IGT, notably improving peripheral insulin sensitivity and preserving or enhancing β-cell function^13–15^. However, they do not effectively address hepatic insulin resistance^3^, which is the key underlying defect responsible for fasting hyperglycemia in individuals with i-IFG^2^.

**Fig. 1:**
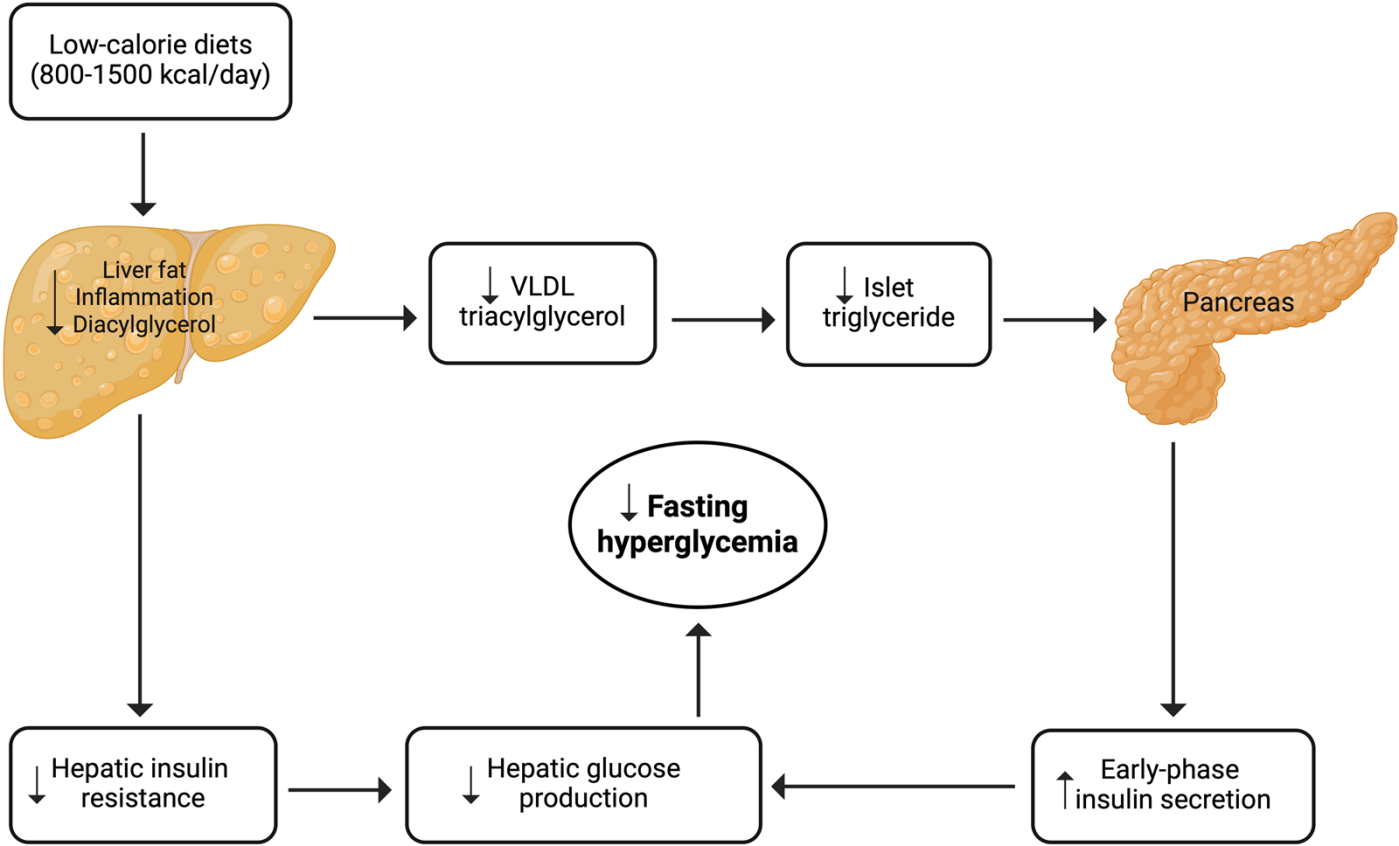
Potential reversal of the twin cycle hypothesis through low-calorie diets in isolated impaired fasting glucose. VLDL very low density lipoprotein.

In recent years, low-calorie diets ranging from 800–1500 kcal/day have gained significant attention in managing type 2 diabetes^8,16–19^. Studies have shown that low-calorie diets can lead to remission and substantial improvements in cardiometabolic risk factors for a significant proportion of individuals with type 2 diabetes^8,16–19^. These diets are generally well-tolerated and safe, with only mild side effects reported. Table 1 summarizes the key low-calorie diet studies conducted in people with type 2 diabetes^8,16–19^. Studies implementing low-calorie diets over a 2–5 month period, primarily high in protein and low in fat, have resulted in a mean weight loss of 7–15 kg (8–15% of initial body weight). This level of weight loss was accompanied by a notable reduction in hepatic fat and improved hepatic insulin sensitivity and first-phase insulin secretion. As a result, fasting plasma glucose levels decreased significantly by 27.8 to 43.2 mg/dL. This suggests that low-calorie diets may also be effective for individuals with i-IFG, as they target the pathophysiological defects characterizing this prediabetes phenotype^8,16–19^. Figure 1 visually depicts the potential reversal of the twin cycle hypothesis through low-calorie diets in individuals with i-IFG. The twin cycle hypothesis^20^ postulates that chronic excess calorie intake results in increased accumulation of fat in the liver, leading to resistance against insulin’s suppression of hepatic glucose production. Additionally, excess liver fat increases lipid transportation to the pancreas, impairing β-cell function and further promoting hepatic glucose production. These self-reinforcing cycles between the liver and pancreas ultimately result  in the onset of hyperglycemia.

**Table 1:** Summary of key low-calorie diet studies in people with type 2 diabetes

Study	Study design & Setting	Study population	Intervention group	Control group	Outcomes
Petersen et al. ^8^	Pre- and Post-intervention study conducted in Yale General Clinical Research Center, USA	8 patients (mean age: 47 [SD: 3] years) with type 2 diabetes and BMI ≥ 30 kg/m^2^	LCD formula (~1200 kcal/day; 50% carbohydrate, 43% protein, 3% fat, 12 g of fiber) for 2 months	None	• Weight loss: 8.0 kg (8.0% of initial weight), *p* < 0.001 • Liver fat content: 81% reduction from baseline (*p* = 0.009) • Hepatic insulin sensitivity: insulin suppression of hepatic glucose output increased from 29% to 93%, *p* = 0.04 • Fasting plasma glucose: reduced by 43.2 mg/dl (from 158.4 mg/dl to 115.2 mg/dl, *p* < 0.001)
DiRECT trial, Lean et al. ^16^, Taylor et al. ^19^	RCT conducted at 46 primary care centers in Scotland and the Tyneside region of England	298 adults (20–65 years) with type 2 diabetes within 6 years of diagnosis and BMI 27–45 kg/m^2^	LCD (825–853 kcal/day; 59% carbohydrate, 13% fat, 26% protein, 2% fiber) intervention for 3–5 months	Routine diabetes care	In the Tyneside cohort (*n* = 58): • Weight loss: 14.8 kg (14.7% of initial weight), *p* < 0.0001 • Liver fat percent: reduced by 127%, *p* < 0.0001 • Early-phase insulin secretion: increased by 0.04 nmol/min/m^2^, *p* < 0.0001 • Fasting plasma glucose: reduced by 27.8 mg/dl, *p* < 0.0001
DIADEM-I trial, Taheri et al. ^18^	RCT conducted in primary care and community settings in Qatar	158 adults (aged 18–50 years) with a short duration (≤3 years) of type 2 diabetes and BMI ≥27.0 kg/m^2^	An LCD formula (800–820 kcal/day; 57% carbohydrate, 14% fat, 26% protein, 3% fiber) for 3 months	Usual diabetes care	• Weight loss: reduced by 12.0 kg (10.3%) in intervention participants and 4.0 kg (4.8%) in control participants (difference: −6.08 kg, 95% CI −8.37, −3.79; *p* < 0.0001) • Insulin sensitivity: improved, as measured by the QUICKI index, by 0.016 points in intervention participants and reduced by 0.006 points in control participants (difference: 0.025, 95% CI 0.015, 0.035; *p* < 0.001)
STANDby trial, Sattar et al. ^17^	RCT conducted in primary care practices in the U.K	25 adults (aged 18–65 years) of South Asian ethnicity with type 2 diabetes for ≤4 years and BMI 25–45 kg/m^2^	An LCD (825–853 kcal/day; 59% carbohydrate, 13% fat, 26% protein, 2% fiber) intervention for 3–5 months	Usual diabetes care	• Weight loss: reduced by 7.2 kg (7.7%) in the intervention group as compared to 0.9 kg (1.2%) in the control group (difference: −6.3 kg, 95% CI −11.0, −1.6; *p* = 0.011) • Fasting plasma glucose: reduced by 18.0 mg/dl in intervention participants and 9.0 mg/dl in control participants (difference: −9.0 mg/dl, 95% CI −34.2, 14.4; *p* = 0.41)

*DiRECT* Diabetes Remission Clinical Trial, *BMI* body mass index, *RCT* randomized controlled trial, *LCD* low-calorie diet, *FPG* fasting plasma glucose, *SD* standard deviation, *CI* confidence interval, *QUICKI* quantitative insulin-sensitivity check index.

The assertion that low-calorie diets could potentially reverse the twin cycle hypothesis in i-IFG is supported by a post-hoc analysis of the PREVIEW (PREVention of diabetes through lifestyle interventions and population studies In Europe and around the World) study, involving 869 individuals (mean age 55.0 years) with overweight (body mass index ≥25 kg/m^2^) and i-IFG^21^. Following an 8-week low-calorie diet phase (810 kcal/day; 41.2% carbohydrate, 43.7% protein, 15.1% fat), the mean weight loss was 10.8 kg (10.7%), with more than four-fifths (82.7%) of participants achieving the targeted weight loss of ≥8%. Notably, the weight loss led to a reduction in mean fasting plasma glucose of 6.5 mg/dl, with slightly over one-third (36.1%) achieving normoglycemia based on fasting plasma glucose alone^21^. The hepatic insulin resistance index significantly decreased by 30%, from 76.69 (SD: 2.31) to 47.42 (SD: 2.41), *p* < 0.001.

Current diabetes prevention guidelines fail to recognize the heterogeneity of prediabetes^22–24^ concerning differences in pathophysiological abnormalities^2,3^ and progression rates to type 2 diabetes among its phenotypes^9^. These guidelines inform the design and development of national diabetes prevention programs that typically deliver standard lifestyle interventions to individuals with any prediabetes phenotype^25–27^. However, recent evidence suggests that standard lifestyle interventions prove ineffective for individuals with i-IFG, while they remain highly effective for those with IGT (with or without IFG) in reducing diabetes incidence^12,28,29^. Therefore, there is an urgent need for further research to identify lifestyle modification strategies tailored specifically to address the distinct pathophysiological defects associated with i-IFG, including investigating the potential efficacy of low-calorie diets.
